# Beyond Immunity: Underappreciated Functions of Intestinal Macrophages

**DOI:** 10.3389/fimmu.2021.749708

**Published:** 2021-09-28

**Authors:** Pailin Chiaranunt, Siu Ling Tai, Louis Ngai, Arthur Mortha

**Affiliations:** Department of Immunology, University of Toronto, Toronto, ON, Canada

**Keywords:** macrophages, monocytes, niche, intestinal, homeostasis, mucosal, macrophage

## Abstract

The gastrointestinal tract hosts the largest compartment of macrophages in the body, where they serve as mediators of host defense and immunity. Seeded in the complex tissue-environment of the gut, an array of both hematopoietic and non-hematopoietic cells forms their immediate neighborhood. Emerging data demonstrate that the functional diversity of intestinal macrophages reaches beyond classical immunity and includes underappreciated non-immune functions. In this review, we discuss recent advances in research on intestinal macrophage heterogeneity, with a particular focus on how non-immune functions of macrophages impact tissue homeostasis and function. We delve into the strategic localization of distinct gut macrophage populations, describe the potential factors that regulate their identity and functional heterogeneity within these locations, and provide open questions that we hope will inspire research dedicated to elucidating a holistic view on macrophage-tissue cell interactions in the body’s largest mucosal organ.

## Introduction

The gastrointestinal (GI) tract presents a unique environment for the local immune system. With its complex tissue structure and constant exposure to dietary and microbial antigens, it is not surprising that the GI tract houses the largest compartment of the immune system, particularly macrophages. Macrophages (MPs) are incredibly heterogenous and versatile members of the innate immune system, serving as mediators of tissue and immune homeostasis. Each organ in the body contains a functionally specialized population of resident MPs that is shaped by the local microenvironment (1). Named for their phagocytic capabilities, MPs have classically been studied for their roles in clearing pathogens and dead cells through phagocytosis.

MPs emerge as one of the earliest immune cells in the developing embryo, and the absence of MP growth factors results in severe morphological defects, alluding to the importance of these cells beyond that of immunity (2–4). In fact, this notion is not new – Élie Metchnikoff’s phagocytosis theory discussed the MP’s role not only in immunity but also in tissue development and embryogenesis (5). In addition to studies of the brain and bone, in recent years, researchers have begun to appreciate these non-immune roles of MPs in other tissues, with particular focus on functions involved in vascularization, nerve growth, and wound healing. The use of genetic fate-tracking systems and high-dimensional transcriptome analysis have revealed how tissue-resident MPs closely interact with stromal, neuronal, and endothelial cells in multiple organs to maintain structural integrity and homeostasis (6–11). Inspired by findings of MP heterogeneity in the liver, lung, and heart, similar non-immune functions are now being elucidated for gut MPs as well (12–14). Here, we review recent advances in MP research in the intestinal tract with a particular focus on murine studies. We summarize the heterogenous ontogeny and functions of gut MPs, delve specifically into their non-immune roles, and propose new directions for the field based on similarities of MP-tissue cell interactions in other organs. Lastly, we discuss implications of these findings in human intestinal disease.

## Ontogeny and Heterogeneity of Intestinal MPs

MPs share many cell surface markers with other myeloid subsets (e.g. CD11b, CD11c or MHCII), thus historically hindering the accurate identification and isolation of these cells from each organ. Recent studies however have allowed for a more complete and nuanced definition of MPs based on the identification of unifying markers across all tissues, the demonstration of developmental pathways with elegant fate-mapping experiments, and the discovery of MP subpopulations using single-cell high-throughput methods.

While intestinal MPs and some subsets of dendritic cells (DCs) express CD11b, CD11c, and MHCII, the additional markers CD64 and F4/80 can be used to reliably identify MPs in steady state tissues (14–16). However, these markers are not as reliable under inflammatory conditions. For example, during pulmonary viral infection, monocyte-derived DCs and inflammatory type 2 classical DCs (cDC2s) in the lung were found to express CD64 (17, 18). Another paper indicated that CD64-expressing MPs can express the DC marker CD103 and CD11b in the steady state, although this apparent expression may be a misinterpretation of the data given the lack of mRNA for CD103 (19). Recent work has demonstrated that intestinal MPs can be specifically characterized by their high expression levels of the chemokine receptor, C-X3-C motif chemokine receptor 1 (CX3CR1). Through genetic ablation of CX3CR1 and its cognate ligand CX3CL1, Medina-Contreras et al. established the receptor’s role in the local self-maintenance of MPs, inhibition of commensal encroachment, and prevention of colitis (20). However, it is now clear that CD11c^+^ MHCII^+^ cells expressing intermediate levels of CX3CR1 represent several populations of mononuclear phagocytes, including differentiating MPs and bona fide CD11b^+^ CD103^-^ DCs (16, 21, 22). Meanwhile, CCR2, the chemokine receptor important for monocyte egress from the bone marrow (BM) and tissue homing in adult mice, is progressively lost over the course of MP differentiation in the majority of MP populations, although it does appear to remain detectable in a subset of MPs, as well as on a subpopulation of CD103^-^CD11b^+^ DCs. In addition to CD64, F4/80, CCR2, and CX3CR1, other, at times more important and appropriate markers for MP identity in the intestine include MerTK and CSF1R (21, 23, 24). However, we would like to advise the reader that careful validation of antibody stains is mandatory when using these marker, as enzymatic-digestion of intestinal tissues for the analysis of MPs may affect binding of antibodies to these additional markers.

The mononuclear phagocyte system (MPS)—a classification system coined by Van Furth et al. in the 1970s to delineate cells of the myeloid lineage—was believed for decades to encompass cells that derive from and are constantly replaced by BM-derived blood-circulating monocytic precursors (25, 26). However, novel fate-mapping models and methods for transcriptomic analysis at the single-cell level have allowed researchers to accurately identify the origin of distinct cell types within the MPS and dissect their ontogeny and function, revealing a refined model of MP development (27). Specifically, these studies identified three distinct waves of hematopoiesis, yielding MPs that seed the developing organs during embryonic, pre-natal, and adult life. These MPs arise *via* (1): primitive hematopoiesis from yolk-sac-derived precursors (2), fetal liver-derived monocytes, and (3) adult definitive hematopoiesis *via* circulating blood monocytes (28, 29). An excellent in-depth review, summarizing the ontogeny and developmental kinetics of tissue-resident MPs across each organ, can be found elsewhere (30). Interestingly, yolk sac- and fetal liver-derived MPs were shown to be long-lived and self-renewing, repopulating the local MP pool during steady state.

**Figure 1:**
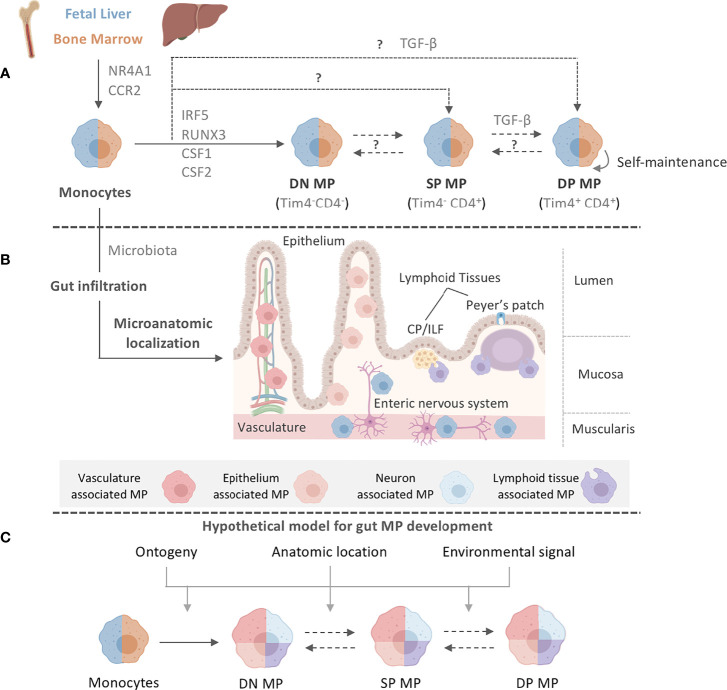
Intestinal MP heterogeneity is determined by distinct ontogeny, transcription profiles, and microanatomic locations. **(A)** Fetal liver monocytes and adult BM-derived monocytes seed the intestinal tract in two distinct waves, giving rise to at least three distinct MP populations in the gut: Tim-4^-^CD4^-^ (DN) MPs, Tim-4^-^CD4^+^ (SP) MPs, and Tim-4^+^CD4^+^ (DP) MPs. Monocytes require CCR2 and NR4A1 for the egress from the BM and differentiation into subsets. Monocytes have been suggested to differentiate into DN MPs in a RUNX3-dependent manner and in an environment rich in CSF1, whereas DP MPs require CSF1 and TGF-β. CSF2 plays a role in gut MP development and impacts the functional and developmental profile of MP, possibly through the actions of IRF5. However, the exact differentiation pathways and plasticity between DN, SP, and DP MPs are yet to be elucidated. The color separation (blue: fetal, light brown: adult) in **(A)** indicates the developmental origin of MPs. Dashed lines indicate potential developmental relationships among gut MPs. It remains unclear if DN, SP and DP MPs constituted a developmental continuum or follow three distinctly developmental pathways. Plasticity between each MP population also remains to be addressed. After birth, infiltration of adult BM-derived monocytes into the intestinal tract requires signals from the microbiome. **(B)** Post infiltration of the gut, MPs accumulate at distinct microanatomic locations across the intestinal tract. Close proximity with characteristic structures allows for the classification of intestinal MPs into vasculature-associated MPs (vMPs, red), epithelium-associated MPs (eMPs, light brown), nerve-associated MPs (nMPs, light blue) and lymphoid tissue-associated MPs (lMPs, violet). Despite their shared monocytic origin, there is evidence of preferential anatomic localization of MPs that has been factored into our current working model for intestinal MP development. **(C)** We propose a working model for gut MP development, in which precursors of distinct developmental origins (blue: fetal, light brown: adult) differentiate into DN, SP, and DP MPs. Colors indicate their accumulation at microanatomic locations like the vasculature (red), the epithelium (light brown), neurons (light blue) or lymphoid tissues (violet). Each MP identity depends on ontogeny, their microanatomic location, and the environmental signals therein. Dashed lines indicate plasticity or direct developmental relationships between DN, SP, and DP MPs.

Intestinal MPs, unlike MPs in most other organs, were believed to lack these self-renewing capabilities and to be completely and constantly replaced by newly gut-infiltrating circulating blood monocytes (**Figure 1A**). The rate of replenishment of gut MPs throughout life is dependent on the presence of the microbiota, a key regulator of intestinal myeloid cells (**Figure 1B**) (31, 32). Differentiation of monocytes to MPs in the lamina propria (LP) follows the classic ‘monocyte waterfall’ pattern, whereby Ly6C^hi^ blood monocytes infiltrate the gut, downregulate the monocytic marker Ly6C, and upregulate MHCII as they mature (16). Further heterogeneity within the seemingly homogeneous MHCII^hi^ Ly6C^-/lo^ MP population was reported in 2018. Shaw et al. identified a population, representing approximately one third of the total mature LP MP pool, of long-lived, self-maintaining, primarily fetal liver-derived MPs in both small intestine (SI) and colon that express CD4 and Tim-4 - the latter a marker also found on long-lived MP populations in other organs, such as the heart (**Figure 1A**) (33, 34). In contrast, Tim-4^-^ CD4^-^ and Tim-4^-^CD4^+^ MPs were rapidly replaced by circulating monocytes throughout life in a microbiota-, CCR2-, and Nr4a1-dependent manner (**Figure 1A**) (33, 35, 36).

Confirming this analysis, fate-tracking of MPs using *Cx3cr1* ^CreERT2^ x *Rosa26*-LSL-YFP mice revealed YFP^+^ MPs in the SI mucosa, submucosa, and muscularis layer even 35 weeks after tamoxifen administration, indicating the existence of long-lived fetal-derived MPs in all microanatomic regions of the gut (8). Interestingly, in these mice, almost half of the MPs within the ileal submucosa and muscularis layers were YFP^+^ at the time of harvest, whereas only 10% of MPs in the ileal mucosa remained YFP^+^, suggesting higher levels of monocyte replacement in the latter region. Tracking of fetal liver-derived monocytes demonstrated an elevated accumulation of pre-natal-emergent MPs in the SI muscularis and submucosa, suggesting a role for these anatomical locations in supporting intestinal MP heterogeneity (8). While LP Tim-4^+^ CD4^+^ MPs are capable of self-renewing locally, subsequent studies using the monocyte-specific fate-mapping model, *Ms4a3* ^Cre^ x *Rosa26*-LSL-tdTomato, paired with transcriptomic analyses of engrafted gut MPs after BM transfer, indicated that even the long-lived Tim-4^+^ CD4^+^ MP population in both the SI and colon is replaced by monocytes emerging through adult definitive hematopoiesis over time (**Figure 1A**) (8, 36). This is in contrast to studies examining the engraftment of adoptively transferred monocytes, which do not express Tim-4 or CD4 at 2 weeks post-transfer (37, 38). An explanation for why self-renewing MPs were overlooked in the past could be the use of the experimental system or time of analysis (37–39). While the selective expression of the diphtheria toxin receptor (DTR) on all CD11c^+^ or CX3CR1^+^ cells allows for the removal of gut MPs, this depletion includes fetal-derived long-lived Tim-4^+^ CD4^+^ MPs. Refinement of this system by targeting the expression of DTR to gut MPs that arise during a distinct hematopoietic wave or by using shielded, instead of total body, irradiation allows for the identification of these long-lived self-sustaining cells (8). Importantly, De Schepper et al. and Liu et al. did not report complete monocyte replacement of all gut MP populations. With 66-74% replacement after 6 weeks and close to 90% after 8 months, self-renewing cells may exist even within the Tim-4^-^ CD4^-^ and Tim-4^-^ CD4^+^ MP populations (8, 36, 40). An additional hypothesis that should be tested is whether monocytes can directly differentiate into Tim-4^+^ CD4^+^ MPs, or whether there is gradual upregulation of *Timd4* or *Cd4* expression in the pool of long-lived gut-resident MPs (**Figure 1A**).

Notably, multiple populations exist within the pool of long-lived Tim-4^+^ CD4^+^ MPs, as recently demonstrated by De Schepper et al. and Kang et al., alluding to location-specific adaptation of these cells (8, 31). Similarly, other markers, such as CD206 or CD11c, have been used to delineate colonic MP populations based on other factors, such as their dependency on the microbiota (31). This heterogeneity has made it extremely difficult to establish a standard nomenclature for intestinal MPs. In order to facilitate the classification of these cells, we have summarized a list of known surface markers, transcription factors, and cytokines/chemokines based on the Tim-4/CD4 categorization of intestinal LP MPs. We advise the reader that this list is by no means exhaustive and should be independently validated (**Table 1**). Our table alludes to differential localization even within each Tim-4/CD4 population based on variable expression of *Lyve1*, *Cd163*, and *Cd206*. Our selection of markers further emphasizes the need for additional study into the transcriptional regulation of each MP identity.

**Table 1:** Heterogeneity of murine **(A)** and human **(B)** monocyte and MPs in the intestinal lamina propria.

A						
		Ly6C^hi^ Mono	Tim-4^-^CD4^-^ MP	Tim-4^-^CD4^+^ MP	Tim-4^+^CD4^+^ MP	
**Surface markers**	*Mertk*	–	+	+	+	
	*Adgre1* (F4/80)	–	+	+	+	
	*Fcgr1* (CD64)	–	+	+	+	
	*Cx3cr1*	+	++	++	++	
	*Mrc1* (CD206)	–	+	+	+	
	*Itgax* (CD11c)	–	+	+	+	
	*Itgam* (CD11b)	++	++	++	++	
	*Ccr2*	++	+	–	–	
	*H2-Ab1* (MHCII)	–	++	+	+	
	*Trem2*	++	+	–	–	
	*Trpv4*	–	–	+	++	
	*Aif1* (Iba1)	–	+	+	++	
	*Adrb1*	–	–	+	++	
	*Lyz1* (Lysozyme)	++	+	–	–	
	*P2ry2*	–	–	+	++	
	*Csf1r*	–	+	++	++	
	*Csf2rb2*	–	+	++	++	
	*Cd209f*	–	–	+	++	
	*Cd209g*	–	–	+	++	
	*Traf1*	–	++	+	–	
	*Adora2a*	++	+	–	–	
	*Adora2b*	++	+	–	–	
	*Tgfbr1*/*Tgfbr2*	–	+	+	++	
	*Il10rb*	–	+	+	+	
	*Vcam1*	–	+	+	++	
**TF**	*Id3*	–	+	–	–	
	*Runx3*	–	++	+	+	
	*Nr4a1*	–	++	+	+	
**Cytokines/**	*Ccl2*	–	++	+	+	
**Chemokines**	*Il1b*	–	++	+	+	
	*Il10*	–	+	+	++	
	*Tnf*	–	++	+	+	
	*Vegfa/Vegfb*	–	+	+	+	
	*Wnt4/Wnt5b*	–	–	+	++	
B						
		CD14+ Mono	Mf1	Mf2	Mf3	Mf4
**Surface markers**	*CCR2*	++	+	+	–	–
	*CX3CR1*	++	++	++	+	+
	*CSF1R*	++	+	+	+	+
	*CD209*	–	+	++	++	++
	*MRC1* (CD206)	–	+	+	+	-/+
	*HLA-DQ/HLA-DR*	–	-/+	++	++	++
	*LAMP2*	+	+	–	–	–
	*IL1R1*	–	+	–	–	–
	*CD163*	++	–	–	+	+
	*CD172A*	+	+	+	+	+
	*ITGAM*	+	+	+	–	+
	*CD64*	++	++	++	+	+
	*CD14*	+	++	+	+	++
	*CD1c*	–	+	++	–	–
	*ITGAX*	-/+	+	+	–	–
**TFs**	*AHR*	–	–	+	++	++
	*RXRA*	++	+	–	–	–
	*TCF7*	–	–	–	+	+
**Cytokines/**	*CCL18*	–	++	+	+	–
**Chemokines**	*IL10*	–	–	–	+	+
	*IL1B*	+	++	+	+	+
	*TNFA*	+	+	+	–	–

Given the anatomical and environmental differences between the small intestine and the colon, it is also important to note that MP heterogeneity exists across gut segments (41). For example, transcriptome analysis demonstrates variations between monocyte graft-derived colonic *versus* ileal MPs, particularly with regards to genes associated with epithelial cell communication and cell metabolism (39). In correlation to the region-specific microbiota abundance, the colon also contains a higher frequency of LP MPs compared to the relatively microbe-scarce duodenum (15). It is important to keep these differences between small *versus* large intestinal MPs in mind, particularly when considering therapeutic approaches, as intestinal pathologies typically present in specific gut segments (39).

## Transcriptional Regulation of Intestinal MPs Within Tissue Niches

The vast heterogeneity of MPs across different organs and microanatomic regions is governed by local tissue microenvironments that produce growth factors, chemokines, and cytokines to drive differential transcriptional programs within these MPs. Mature tissue-resident MPs can transcriptionally adapt to changes in their environment, as illustrated in an elegant study by Lavin et al. Here, peritoneal MPs partially adapt gene expression signatures characteristic of alveolar MPs upon adoptive transfer into the lung (1). Interestingly, van de Laar et al. refined this picture and demonstrated that precursors from all three hematopoietic waves (embryonic, fetal, and adult) were capable of adopting the phenotype of an adult alveolar MP when transferred into an empty niche. In contrast to Lavin et al., they demonstrated that MPs isolated from the liver and colon were unable to engraft into the lung. Even though engraftment of MPs from the peritoneum was observed in the lung, these cells were incapable of fully adopting an alveolar MP phenotype. Notably, Lavin et al. performed adoptive transfer of peritoneal MPs into conventional C57Bl/6 mice (already containing a local pool of alveolar MPs), while van de Laar et al. transferred peritoneal MPs into *Csf2rb^-/-^* hosts (lacking all alveolar MPs). The results of these reports either suggest the presence of a peritoneal precursor population, an adaptable differentiation program specifically in peritoneal MPs, or the requirement of a pre-existing pool of alveolar MPs to initiate the reprogramming of mature infiltrating cells (42). A co-transfer of alveolar MPs or their precursors along with congenic peritoneal MPs into *Csf2rb^-/-^* hosts could help to validate these interpretations, at least in parts. An intriguing study led by Li et al. revealed that fetal liver-derived monocytes have a superior capacity to replace alveolar MPs in postnatal *Csf2ra^-/-^* hosts when compared to primitive, yolk-sac-derived MPs. An underlying transcriptional difference and increased metabolic fitness of fetal liver monocytes may possibly be the result of elevated CSF2RA expression on these precursors. Collectively, these findings emphasize the need to improve our understanding of both the necessary growth factors and the transcriptional regulators of MP identity across organs and their microanatomic locations to uncover the true complexity of MP heterogeneity.

As with all MPs, intestinal MPs express a unique transcriptional program that dictates their core MP characteristics, such as phagocytosis and antigen sampling. For example, PU.1 has long been studied as a master regulator of MP lineage specification and commitment (43, 44). Interferon regulatory factor 8 (IRF8), another pivotal transcriptional factor in adult myeloid lineage commitment and inflammatory response, plays an additional role for the maturation of embryonically derived MPs (45–48). CCAAT/enhancer-binding protein β (C/EBPβ) has also been shown to be essential in the pro-inflammatory fate of MPs (49, 50). Importantly, PU.1 and C/EBPβ induce the upregulation of colony stimulating factor 1 receptor (CSF1R) *via* a two-step chromatin remodeling process in MP precursors as they differentiate (51). This mechanism results in a positive feedback loop that amplifies the cell’s responsiveness to CSF1 or IL-34 - growth factors required for MP differentiation and survival (52–54). Both CSF1 and IL-34 act through CSF1R, but display distinct patterns of expression. IL-34 has been reported to be secreted in the brain, eye, skin, and peripheral nerves, while CSF1 shows a far broader expression across the body (52–54). Interestingly, CSF1 has been shown to induce PU.1 expression in hematopoietic stem cells (HSCs), suggesting the possibility of a growth factor-transcription factor feedback loop to further drive MP differentiation (54). The necessity of CSF1R in MP development and survival has been established using genetic mouse models and antibody-mediated neutralization of CSF1. In line with this, *Csf1r* ^-/-^ and *Csf1* ^op/op^ mice show significantly reduced MP populations, with recognizable differences in microglia and Langerhans cell numbers in *Csf1r^-/-^ versus Csf1^op/op^* mice (3). Injections of neutralizing anti-CSF1 antibodies abrogate the development of tissue-resident MPs, with prolonged treatment resulting in an almost complete loss of MPs even in the gut (3, 55, 56).

Similar to the brain and lung, an indispensable factor of monocyte differentiation in the intestine is transforming growth factor-β (TGF-β) (24, 57–59). With regards to MPs, TGF-β has long been associated with an anti-inflammatory wound-healing phenotype as these cells have been shown to secrete the cytokine in models of chronic pulmonary fibrosis and skin wounds (60, 61). In the gut, TGF-β—particularly the isoform TGF-β1—plays an extensive role in regulating both local adaptive and innate immune responses to induce oral tolerance, and much of its prevalence in the tissue depends on an intact microbiota (62–64). Given its ubiquitous role, it is likely that the multiple sources of TGF-β, including intestinal epithelial cells, DCs, and MPs themselves, may all affect gut-seeding monocytes during their differentiation process (62, 65, 66).

As monocytes transition into MPs in the colon, genes associated with the TGF-β receptor (TGF-βR) signaling pathway, such as *Smad7* and *Tgfbr1/2*, were shown to be highly upregulated (24). The same study demonstrated that the increased expression of intestinal MP-specific genes, such as *Cx3cr1*, *Il10*, and *Itgax*, requires TGF-βR signaling (24). Notably, TGF-β signaling seems to induce differential gene expression profiles in intestinal CD11c^+^ cells, containing DCs and MPs, *versus* those in the brain or lung, alluding to the multiplicity of other extracellular factors in each organ that concurrently shape MP identity (24, 57, 59). The necessity of TGF-β signaling on intestinal MPs has primarily been studied in mouse strains with TGF-βR-truncated or -dysfunctional CD11c^+^ cells. For example, mice containing MPs with truncated TGF-βR experience more severe DSS-induced colitis, with reduced IL-10 production and delayed goblet cell regeneration, demonstrating the role of TGF-β in promoting MP-mediated immunosuppression (67). As will be discussed shortly, a unique characteristic of intestinal MPs is their hyporesponsiveness to exogenous stimulation. However, this suppressive phenotype does not seem to depend on TGF-β signaling but instead on IL-10 signaling, with both pathways imprinting non-overlapping features of colonic MPs. Instead, mice with TGF-βR-deficient CD11c^+^ cells displayed elevated numbers of immature Ly6C^hi^MHCII^lo^ colonic MPs and higher *Ccl8* expression in mature MPs, suggesting that the TGF-β-TGF-βR axis may aid in monocyte recruitment and MP turnover (24).

Another interesting transcription factor that predominantly regulates the differentiation program of monocytes and MPs upon TLR engagement is interferon regulator factor 5 (IRF5). IRF5 is strongly upregulated upon LPS stimulation of human blood monocyte-derived MPs, resulting in elevated production of TNFα, IL-1β, IL-12, and IL-23 while repressing IL-10 (68). These activated MPs preferentially polarized CD4^+^ T cells towards a Th1/Th17 phenotype and promoted inflammation in several autoimmune disorders (69). In line with these findings, the absence of *Irf5* in MPs ameliorated disease in two microbiota-driven models of colitis due to a decrease in infiltrating Ly6C^hi^ MHCII^+^ monocytes (70). Notably, *Cx3cr1^Cre^* x *Irf5^flox/flox^* mice revealed an apoptosis-independent defect in the colonic MP compartment even in the absence of inflammation, implicating the importance of this transcription factor during normal gut MP development (70).

Transcriptome analysis of tissue-resident MPs across organs identified gut-specific expression of Runt-related transcription factor 3 (RUNX3) (**Figure 1A**) (1). RUNX3 expression has been shown to be driven by TGF-β in colonic MPs (24, 71). In line with this, *Runx3* ^-/-^ mice show impaired differentiation of anti-inflammatory MPs and cDC2s and consequently develop spontaneous colitis (72). Recent work by Scott et al. identified the transcription factor, zinc finger E-box binding homeobox 2 (ZEB2), to be required for maintaining tissue-specific identities of MPs in the liver, lung, brain, spleen, and gut (73). Analogous to RUNX3, TGF-β treatment enhances ZEB2 expression in MPs in the context of human hepatocellular carcinoma, suggesting possible co-regulation of both transcription factors through TGF-β-mediated signaling (74).

Notably, transcriptional heterogeneity between monocyte- and fetal-derived intestinal MPs extends beyond their ontogeny and alludes to environmental influences. To study the effects of long-term MP residency in the gut, De Schepper et al. labeled adult intestinal MPs in *Cx3cr1* ^CreERT2^ x *Rosa26*-LSL-YFP mice and compared the transcriptional signatures of YFP^-^ monocyte-replaced MPs *versus* YFP^+^ self-maintaining MPs in the SI and colonic LP 35 weeks later (8). Self-maintaining MPs were enriched in genes involved in homeostatic tissue maintenance, including epithelial cell and neuronal differentiation and angiogenesis, suggesting that self-maintaining, Tim4^+^CD4^+^ MPs contribute to tissue homeostasis during steady state (8). In the peritoneal cavity, resident MPs were found to require the transcription factors KLF2 and KLF4 for their *Timd4* expression and consequent function of apoptotic cell clearance—whether this applies to intestinal Tim-4^+^ MPs remains to be seen (75). On the contrary, MPs that were replaced by adult BM-derived monocytes expressed genes known to regulate innate immune responses and phagocytosis. Indeed, monocyte-derived Tim-4^-^ CD4^-^ MPs were found to express genes distinct from long-lived Tim-4^+^ CD4^+^ MPs, including those involved in cytokine production and phagocytosis (e.g. *Stat4*, *Slamf7*, and *Traf1*) (33). Interestingly, these Tim-4^-^ MPs also express inhibitor of DNA binding 3 (ID3), a TGF-β-responsive transcriptional repressor that, by contrast, serves as a critical regulator of fetal-derived Kupffer cell development (7, 76). This not only reflects the TGF-β dependency of MP populations across organs, but also suggests that the same cytokine can induce either monocyte- or fetal-derived MP identity depending on the context (**Figure 1A**).

Interestingly, a small proportion of monocytes can differentiate into Tim-4^+^ self-maintaining MPs, both during steady state or upon MP depletion and gut inflammation (8, 32, 33, 36–38). These monocytes give rise to fully mature tissue-resident MPs that partially recapitulate the gene expression profile of fetal-derived MPs (8, 39). Whether or not their prolonged residence in the gut will enable these cells to eventually fully adopt the gene expression signature of fetal-derived long-lived MPs over time remains to be elucidated (**Figure 1C**). The determining factors of monocyte differentiation into gut-resident MPs remain unknown, although a recent study on peritoneal MPs suggests that monocytes acquire a mature tissue-resident identity when entering an empty niche, wherein the original resident MPs have been ablated (77). Notably, the presence of long-lived Tim-4^+^ MPs across other organs suggests *Timd4* expression may reflect the duration of residence within the tissue and not necessarily MP ontogeny (78). Thus, in the context of intestinal MPs, we assert that these transcriptional complexities call for careful characterization and definition of MP populations based more on their function and localization than merely on their ontogeny.

Indeed, the heterogenous transcriptional signatures of intestinal MPs reflects the differences in their local microenvironments and functional specializations. For example, distinct MP populations can be defined in the gut based on their expression of genes implicated in either epithelial, neuronal, or vascular homeostasis. The physical proximity to non-hematopoietic cells across the gut suggest that MPs adapt these specialized transcriptional programs to support their neighboring cells (8). Guilliams et al. reported a similar regional specialization of MPs in the liver (7, 9). MP interactions with other long-lived, tissue-resident cells, like innate lymphoid cells (ILCs), also shape regional MP identity. For instance, lung ILCs and the lung epithelium produce high levels of colony stimulating factor 2 (CSF2), a growth factor involved in differentiation and homeostasis of alveolar MPs (79–81). Gut MP homeostasis partially depends on CSF2, which is most abundant within intestinal cryptopatches (CPs) and isolated lymphoid follicles (ILFs) (82). Since *Csf2* ^-/-^ mice display only a partial reduction in intestinal MPs, the aforementioned lymphoid structures, which contain group 3 ILCs (ILC3s) and MPs, may serve as a niche for a unique CSF2-dependent MP population (82–84). Refining the map of myeloid growth factors across the intestinal tract will help to identify niches that support the maintenance and transcriptional adaptation of MPs in the gut.

## MP Recognition of the Intestinal Microbiome

As their name suggests, MPs are best known for their proficient phagocytic abilities, particularly in the context of engulfing microbes for innate host defense. Given that the intestinal tract hosts the most diverse microflora in the body, it may seem surprising that gut MPs are not constantly activated and secreting proinflammatory cytokines. Since MP-microbe interactions are a classic immune function of intestinal MPs, we will only briefly summarize how MPs respond to the microbiota and contribute to immune homeostasis.

### MPs, Luminal Microbes and Gut Immune Homeostasis

To detect and mediate responses to the microbiota, MPs are equipped with an array of sensory pattern-recognition receptors (PRR) (85). The mechanisms behind PRR regulation in intestinal MPs have been extensively studied and discussed over the past decade; we encourage our readers to seek reviews on this topic (86, 87). Inflammasomes, particularly NLRP3 and NLRC4, were found to play important roles in inducing IL-1β-dependent, monocyte and MP-driven type III immune responses following bacteria commensal and pathogen encounter (88–92). Similarly, CX3CR1^+^ cells, including subsets of DCs, are implicated in initiating specific anti-fungal programs to promote pathogen clearance (93–96). Together, these data suggest that microbe-initiated activation of inflammasomes in monocytes, MPs and DCs can result in a context-dependent immune response. While there are multiple mechanisms of microbial uptake and recognition, the exact contributions of monocytes, MPs and DCs remain controversial, and distinction of these cell types should be considered when interpreting experiments centered around antigen sampling and microbial regulation (97–99). Persson et al. provide a detailed discussion on mechanisms of antigen sampling by DC and MPs in the gut (100).

Cytokine secretion and activation of MPs by the microbiota has been suggested and was found to promote the constitutive secretion of IL-10 by SI and colonic MPs in support of oral tolerance and regional abrogation of inflammatory disease (98, 101, 102). Some of the microbiota-dependent mechanisms proposed for gut MP secretion of IL-10 include the attachment of commensal microbes to the gut epithelium, the colonization with helminths, or the secretion of IL-4 and IL-5 by T helper type 2 (Th2) cells (103). Without a doubt, MP-derived IL-10 contributes to several homeostatic and anti-inflammatory processes, including the modulation of intestinal epithelial healing and the host immune response promoting local regulatory T (T_reg_) cell expansion (15, 101, 104–107). Varying levels of IL-10 were observed in colonic, but not SI, MPs of mice colonized with different microbiota, suggesting that the tolerogenic function of MPs relies on regional differences in environmental factors, including the microbiota (101, 106). Collectively, these data suggest an instructive role for commensals and pathogens alike in shaping local MP development and function, leaving several questions on the involvement of non-bacterial commensal in the regulation of MP functions unexplored (108–110).

**Figure 2:**
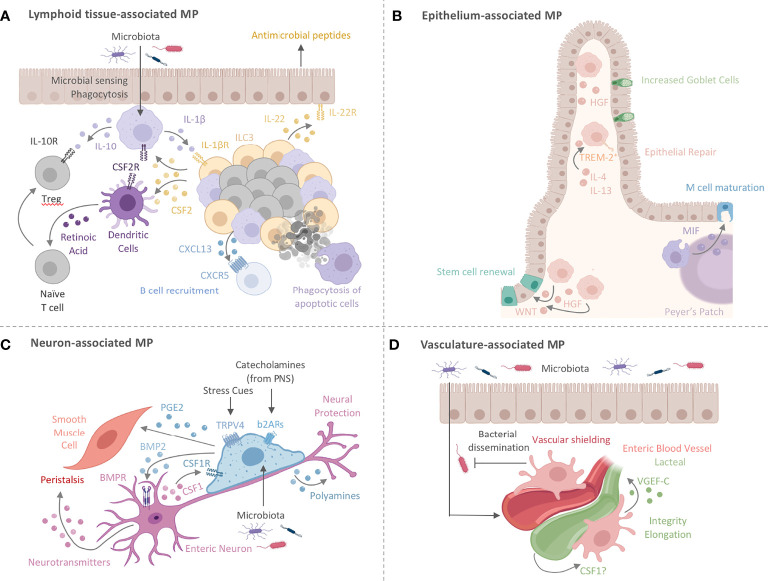
Intestinal MPs play multiple non-immune roles to maintain tissue homeostasis. **(A)** Lymphoid tissue -associated MPs (lMPs) accumulate in PPs, ILFs and CPs. These lMPs sense and sample microbes and surround the B cell follicles within PPs and ILFs. Microbial recognition by lMPs facilitates the release of IL-1β and activates lymphoid tissue-resident group 3 innate lymphoid cells (ILC3s). ILC3s release CSF2 and IL-22 to engage CSF2R on myeloid cells or IL-22R on epithelial cells. The latter is prominent in facilitating antimicrobial activity. The former (CSF2-CSF2R) acts on DCs and lMPs to induce the production of IL-10 and RA, both critical in facilitating the conversion of naïve T cells in to regulatory T cells (Treg). Lymphoid tissue -associated MPs have also been shown to release the B cell-recruiting chemokine CXCL13 and clear apoptotic B cells resulting from failed somatic hypermutation or class-switch recombination, thus contributing to the local IgA response. **(B)** MPs lining the epithelium (eMPs) near intestinal crypts induce stem cell renewal by inducing WNT signaling in intestinal epithelial stem cells (IESC). Epithelium-associated MPs adopt an alternative activation phenotype when stimulated with by IL-4 and IL-13, by upregulating TREM-2 and aiding in epithelial repair and goblet cell proliferation. MPs in the subepithelial dome of the PPs have been shown to induce M cell maturation through the release of MIF. Hepatocyte growth factor (HGF) is another protein that mediates epithelial repair and is possibly produced by eMPs post injury with likely varying locations around the crypt and the villus. **(C)** Nerve-associated MPs (nMPs) in the intestinal muscularis layer are stimulated by the gut microbiota and regulate peristalsis *via* BMP2-mediated activation of enteric neurons. Enteric neurons release neurotransmitters to induce smooth muscle cell contractions. Direct activation of smooth muscle cell contraction is mediated by nMP-release of PGE2. In addition, nMPs release polyamines in response to signals from the microbiome, catecholamines, and other stress cues to facilitate neuronal protection. In turn, enteric neurons secrete CSF1 to support nMP survival. **(D)** Across the lamina propria, vasculature-associated MPs (vMPs) wrap around blood vessels to aid in angiogenesis, lipid transport, dead cell clearance, vessel integrity and elongation. In the presence of an intact microbiota, these MPs are rapidly replaced by monocyte-derived cells to induce vascular repair while protecting against bacterial dissemination upon injury and infection. How the endothelium in turn maintains MP survival is currently unknown. Vasculature-associated MPs in close proximity to small intestinal lacteals facilitate vessel elongation and integrity through the microbiota-driven production of VGEF-C.

Although the microbiota is required for proper MP function and intestinal health, it also requires tight control by the host innate immune system. In fact, MPs, DCs and intestinal barrier dysfunction have been shown to impair oral tolerance and promote inflammation and disease, as implicated in the pathogenesis of Crohn’s disease (CD) (111). Genetic ablation of CX3CR1, CSF-2, or IL-10 in mice results in a loss of oral tolerance towards luminal antigens, consequently increasing susceptibility to intestinal inflammation (82, 101, 103, 112). For instance, mice deficient in IL-10 develop spontaneous colitis through an aberrant response to commensals (113, 114). This is supported by data in humans, where genetic mutations in the IL-10 receptor (IL-10R) were shown to be associated with the development of severe pediatric IBD (115). Specifically, IL-10R signaling was reported to be critical for gut MPs to abrogate colitis through the activation of STAT3 (116, 117). A subsequent study demonstrated that selective IL-10R-deficiency in MPs drives intestinal inflammation through uncontrolled IL-22-dependent activation of IECs and the subsequent recruitment of inflammatory neutrophils (118). Notably, *Il10* deletion in colonic MPs does not perturb homeostasis, suggesting that the inflammatory phenotype seen in *Il10* ^-/-^ mice is independent of MP-derived IL-10. These findings further confirm the requirement for T_reg_-derived IL-10 in dampening inflammation at mucosal surfaces, suggesting a possible interaction between T_regs_ and intestinal MPs (112, 119). Zigmond et al. ’s study indicates that the MP responsiveness to IL-10 is critical in preventing spontaneous colitis. Thus, these data together allude to the necessity of the IL-10-IL-10R axis for both MPs and Tregs in maintaining local immune tolerance (112, 119). While the IL-10-IL-10R interaction sustains Treg and MP homeostasis, intriguing new data suggest that intestinal MPs may also contribute to the induction of immunomodulatory and barrier-supporting IgA through the production of the B cell-recruiting chemoattractant CXCL13 and the release of cytokines involved in class-switch recombination, suggesting a possible role for MPs in humoral immunity at mucosal surfaces (**Figure 2A**) (84, 103).

Crosstalk between MPs and type 3 innate lymphoid cells (ILC3s) in ILFs offers yet another mechanism for tolerance induction and host defense against enteric infections. In the steady state, microbiota-driven IL-1β production by colonic MPs promotes CSF2 release by ILC3s, which in turn activates IL-10 and retinoic acid (RA) secretion in MPs and DCs (82). These activated ILC3s also produce IL-22, which maintains barrier integrity and induces antimicrobial peptide secretion by epithelial cells (120). These interactions have been shown to be critical for the induction of oral tolerance and defense against attaching/effacing enteric pathogens such as *Citrobacter rodentium* (82, 120–123). Given the importance of CSF2 in maintaining monocytes and MPs, it is likely that the MP-ILC3 crosstalk in colonic ILFs may also serve as a local site for the growth and differentiation of monocyte-derived MPs (83). Importantly, the combination of proinflammatory IL-1β-secreting MPs and immunomodulatory IL-10-producing MPs in this crosstalk further emphasizes the complexity and heterogeneity of intestinal MPs in facilitating intestinal immune and tissue homeostasis (**Figure 2A**).

Taken together, the development and function of MPs have profound consequences on intestinal health. It is evident that the location of MPs within an organ is critical to their identity, as microenvironmental cues from neighboring cells imprint functional programs tailored to serve the local microanatomic niche (**Figure 1B**) (124). Additional MP-immune interactions and classical contribution to the regulation of intestinal immune homeostasis have been excellently reviewed elsewhere (86). However, the interactions of MPs and non-hematopoietic cells in the gut is an expanding area of research and we will therefore focus on intestinal MP functions going beyond their traditional roles by summarizing recent progress on this new frontier of MP non-hematopoietic cell interactions.

## Intestinal MPs Go Beyond Immunity

**Figure 3:**
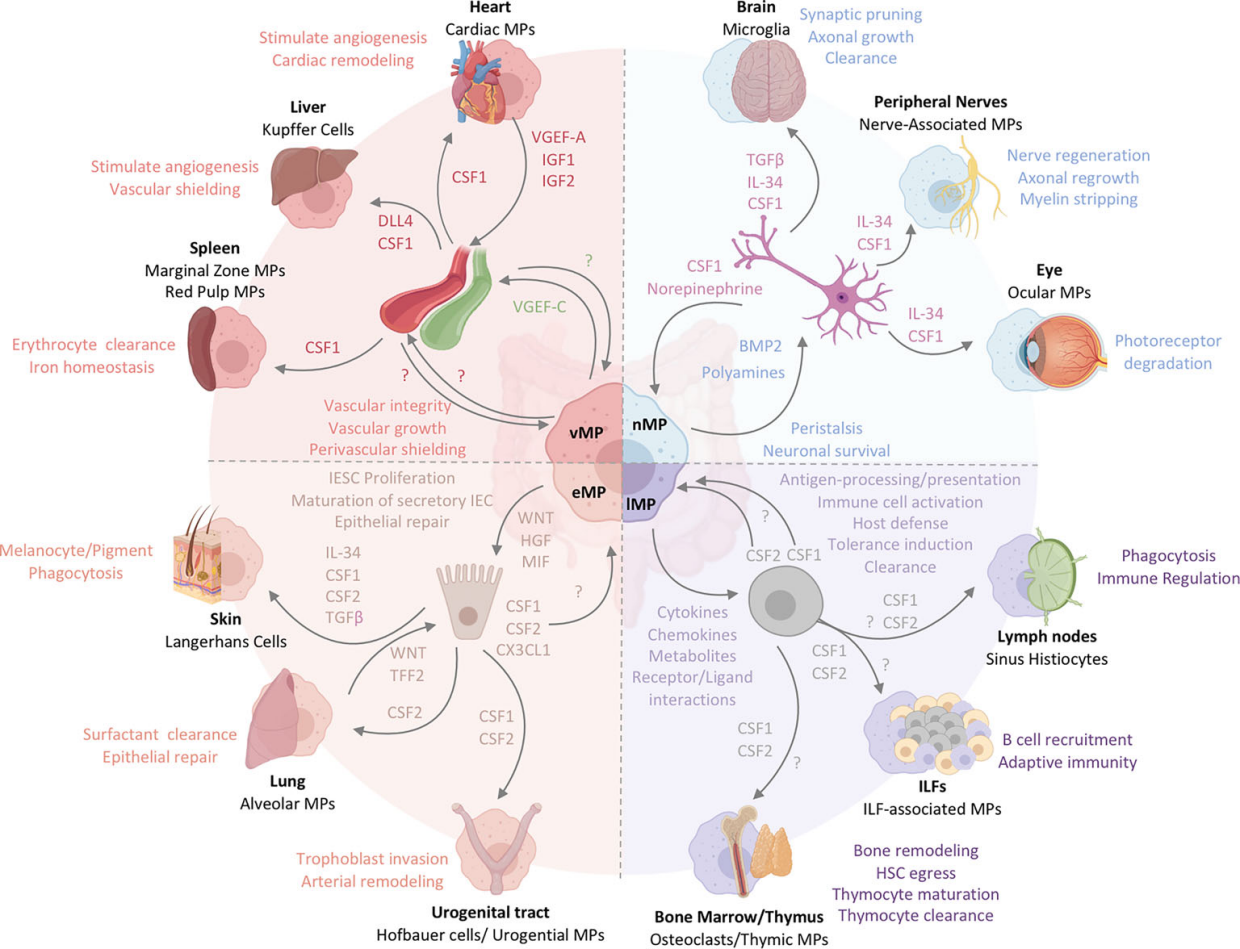
The functional heterogeneity of intestinal MPs mirrors the multifaceted roles of tissue-resident MPs across organs. Intestinal MPs maintain tissue homeostasis through their interactions with neurons, immune cells, epithelial cells, and endothelial cells. These functions are dependent upon the associations with both immune and non-hematopoietic cells within their respective microenvironments. Released factors and physiological consequences of vasculature-associated MPs (vMPs, red), epithelium-associated MPs (eMPs, light brown), nerve-associated MPs (nMPs, light blue) and lymphoid tissue-associated MPs (lMPs, violet) are indicated by their respective colors. Arrows indicate the source of a given regulator (i.e. produced by MPs perceived by neighboring cell). Nerve-associated MPs in the brain, peripheral nervous system or the eye have specialized functions tailored to their location. These cells depend on neuron-released TGF-β, IL-34 and CSF1. Whether intestinal nMPs require the full spectrum of growth factors is unknown. In addition, a role for maintaining sensory neurons, neuronal growth or synaptic pruning in the gut remains to be shown. Lymphoid tissue-associated MPs are vital in antigen sampling, processing and presentation and mediate dead cell clearance of leukocytes within gut-associated lymphoid tissues. They contribute a plethora of cytokines, chemokines, metabolites and receptor ligands which plays an essential role in host defense against microbes, the orchestration of tolerance induction, and immune cell activation. CSF2, produced by T cells and ILC3s, is essential in facilitating most of these processes in the intestinal tract. It remains to be shown whether CSF1 or other myeloid growth factors like IL-10 support their biological relevance in intestinal immune homeostasis. Peripheral lymph node- and ILF-associated MPs mirror the function of lMPs in the gut, likely due to the similarities in the development and architecture of these organs. The thymus and BM, primary lymphoid tissues, host MPs that mediate tissue remodeling and control the hematopoietic stem cell egress, while also facilitating T cell maturation and clearance. Whether lMPs in the gut are capable of these functions remains to be addressed. The interactions of epithelium and MPs in the gut support IESC proliferation, maturation of secretory IEC and the repair the of damaged epithelial monolayer. CX3CL1 controls the adaptation of an eMPs phenotype but it remains to be shown if CSF1 or CSF2 produced by IECs in the gut contribute to this identity. In contrast to the gut, Keratinocytes, alveolar type 2 cells and urogenital epithelial cells produce distinct combinations of myeloid growth factors that facilitate development and functional specialization of MPs in their respective environments. In the case of the lung, WNT-release by lung macrophages mirrors the mechanism used by intestinal eMPs to facilitate epithelial repair. Functions of eMPs collectively promote tissue homeostasis and tissue remodeling. While the small intestinal lymphatic system is supported by vMPs through the production of VGEF-C to mediate vessel integrity and elongation, molecular mechanisms allowing for a deeper understanding of vMP-blood vessel interactions are currently not known for the intestinal tract. Vasculature-associated MPs surrounding intestinal blood vessel in a symmetric pattern that shields the endothelium from the environment, serving as a cellular firewall for the prevention of microbial spread into neighboring organs. Filter and barrier functions have been assigned to MPs in the spleen and the liver, where these cells are supported by endothelium-derived growth and differentiation factors. Cardiac MPs in fact receive survival signals from the endothelium *via* the release of CSF1 and in turn support the release of insulin growth factor (IGF) 1 and 2 as well as VGEF-A for cardiac remodeling and angiogenesis.

Damage to the gut during acute and chronic inflammation has immediate and long-lasting effects on the organ’s physiology, reaching beyond both transient and permanent activation of hematopoietic immune cells. The changes in the hematopoietic compartment of an organ are only a fraction of the complete response during inflammation. The adaptations of non-hematopoietic cells during inflammation and repair are beginning to gain their well-deserved attention in assembling a holistic, organ-wide immune response (125–128). Tissue cells show significant and long-lasting changes during development, damage, inflammation, or repair, raising the possibility of tissue cell-MP interactions that may permanently alter intestinal homeostasis (125). MPs collaborate with non-hematopoietic cells to actively remodel tissues under homeostatic and inflammatory conditions and in turn, are nurtured by the growth and survival factors secreted by their non-hematopoietic neighbors. The environmental and anatomical complexity of the intestine, paired with a seemingly arbitrary distribution of MPs across the gut’s microanatomic locations had hindered the identification of obvious tissue cell-MP liaisons (**Figure 1B**). Paradigm-setting reports on tissue cell-MP interactions in the skin, brain, and liver, however, have provided roadmaps on how to tackle this problem in the intestine. These studies identify unique and critical roles for specific MPs in sustaining and shaping local tissue cells *via* interactions that go beyond classical immunity (**Figure 1B**) (8, 24, 33). In the following sections, we summarize our current understanding of these non-immune functions of intestinal MPs, highlight the importance of the local tissue microenvironment in shaping these interactions, and share outstanding questions. We then superimpose gut-specific MP-tissue interactions with those reported in other organs to inspire an overarching understanding of MP-tissue cell interactions (**Figures 2** and **3**).

### Intestinal MPs Fortify the Epithelial Barrier

Intestinal epithelial cells (IECs), forming a monolayer barrier between the LP and lumen, are the first physical line of defense in the protection against commensal and pathogenic microbes. MPs line the intestinal epithelium, and emerging data have indicated that MPs aid these cells in their function and development. Steady state MPs constitutively express low levels of TNFα, which has been shown to regulate enterocyte growth, maintain epithelial barrier integrity, and stimulate the production of tissue remodeling proteins in intestinal mesenchymal cells (23, 129, 130). The secretion of soluble factors, such as the lipid mediator prostaglandin E_2_ (PGE_2_) by MPs, further supports the epithelial barrier (131). Given the vast surface of the epithelium and the regional delineation of different epithelial cell types, these MP-tissue cell interactions may serve different functions depending on location. For example, interactions where MPs aid in epithelial stem cell repair may most likely be observed at the crypts of the epithelium, while MPs targeted to support enterocytes or other differentiated epithelial cells may execute their functions in the upper regions of the LP and the villus. The transient ablation of gut MPs through anti-CSF1R antibody treatment for example disrupts the proliferation of intestinal stem cells, resulting in reduced expression of the WNT receptor *Lgr5* on intestinal epithelial stem cells, and impair maturation of Paneth cells across the SI (55). Depletion of MPs further indirectly blocks M cell differentiation in favor of goblet cells, thereby potentially affecting the transcytosis of luminal particles across the intestinal wall (55). Defining the interaction of MPs and IEC should therefore be seen in a multicontextual setting, accounting for the microanatomic localization of MPs, their functional specialization and effects on the epithelial layer

It is in cases of intestinal insult that these epithelium-associated MPs (eMPs), found along the crypts and villi of the LP, become truly critical for repair. For example, following tissue damage, MPs around intestinal crypts were responsible for the proliferation and survival of epithelial progenitor cells in a MyD88-dependent manner (132–134). In a murine model of acute colonic epithelial injury, Seno et al. demonstrated increased infiltration of triggering receptor expressed on myeloid cells 2 (TREM-2)^+^ MPs around injured areas. TREM-2 promotes repair genes in IL-4- and IL-13-stimulated, alternatively activated MPs by dampening NFκB signaling and thus facilitates wound closure and epithelial proliferation and repair (**Figure 2B**) (135). On the other hand, colonic MP expression of TREM-1 is associated with colitis, and blocking TREM-1 abrogates local levels of monocyte chemoattractants and pro-inflammatory cytokines. Tripartite motif-containing 33 (TRIM33) is a cell surface protein that plays a role in inducing an immunomodulatory MP phenotype. For example, *Trim33* ^-/-^ mice contain monocytes that do not differentiate into colonic MPs and display impaired resolution of colonic inflammation in DSS-induced colitis (136, 137).

Another role for eMPs in epithelial repair was found by a different group, who reported CD206^+^ MPs in close proximity to the damaged mucosa of ulcerative colitis (UC) patients. These MPs expressed elevated levels of *Wnt1* and *Wnt3a* compared to pro-inflammatory MP populations and activated the reparative WNT-β-catenin pathway in IECs (**Figure 2B**). Co-culturing IECs with alternatively activated murine MPs increased their levels of nuclear β-catenin through a STAT6-dependent pathway from MPs (138, 139). Conversely, deletion of STAT6 in epithelial cells drastically impaired CD206^+^ MP numbers in the colonic LP, altered epithelial repair, and exacerbated tissue damage, suggesting a possible STAT6-dependent circuit for both epithelial cell proliferation and MP activation (140). Hepatocyte growth factor (HGF) is growth factor that aids in the eMP-IEC interactions and was reported to be produced by human and murine MPs to promote the growth of epithelial cell lines *in vitro*, independent of direct MP-epithelial cell contact. The epithelial intrinsic effects and actions of HGF were not investigated in this report. However, MP-derived HGF may utilize its receptor c-MET and its putative co-receptor CD44v, both targets of WNT signaling and expressed on IESC, to facilitate epithelial repair (141). Notably, c-MET deficient epithelial cells developed normally during steady state but failed to regenerate at rates comparable to c-MET sufficient epithelial cells (141). Interestingly, depletion of *Porcn*, a gene essential for the synthesis and secretion of WNT ligands, in intestinal MPs resulted in hypersensitivity to radiation-induced injury and delayed repair of intestinal stem cells, suggesting MP-derived WNT ligands are critical regulators of epithelial repair (**Figure 2B**) (142). It remains to be shown whether MP-derived WNT secretion precedes the beneficial effects of HGF in promoting epithelial repair (143).

HGF is not the only factor regulating WNT’s actions on the intestinal epithelium. Concerted actions of WNT, bone morphogenetic protein (BMP) and Notch maintain the crypt-villus axis (144–146). Prompted by evidence that MPs in the muscularis layer express BMP2 to aid in enteric neuron maintenance, it remains possible that eMPs contribute to regulating epithelial cell differentiation through BMP production (147). Within the epithelium, BMP2 and BMP4 make up the main ligands that drive intestinal BMP signaling. Opposing WNT signaling, BMPs promote the differentiation of crypt-residing IECs into the secretory lineage further evident by the development of polyposis in the stem cell zone upon conditional deletion of the BMP receptor 1A (BMPR1A) in the epithelium (148–150). To maintain a stem cell niche, crypt-surrounding myofibroblasts and smooth muscle cells were shown to produce BMP inhibitors, such as Noggin or Chordin-like 1, generating opposing gradients of high WNT and BMPs along the crypt-villus axis, respectively (149, 151, 152). The contribution of MPs in shaping the WNT/BMP-gradient remains an intriguing possibility and requires further research dissecting the MP-specific release of BMPs across microanatomic regions of the intestine. Considering the steady-state regulation of BMP production in muscularis macrophages through the TLR-Myd88 axis, BMP production by MPs in the LP may follow a similar pathway and could possibly counterbalance MP-produced WNT (147). WNT expression was found to be enriched in SI-resident MPs when compared to monocytes, while BMP expression appeared highest in Tim-4^+^CD4^+^ MPs, suggesting a functional specialization of MP subsets (33). However, it remains unknown whether WNT and BMPs are co-produced by specific MP subsets or exclusively produced by MPs within specific microanatomic regions.

WNT signaling regulates rapid intestinal epithelial renewal, with the entire epithelium being completely replaced every 4-5 days. As apoptotic IECs are shed off at the tip of the villi, they are phagocytosed by closely situated MPs and DCs. Removal of dead IECs by eMPs supports barrier homeostasis under both steady state and inflammatory conditions (19, 153). This engulfment of apoptotic cells has been shown to alter MP identity, presenting an intriguing aspect of how the epithelium can shape their associated MPs. These transcriptional changes, reported by Cummings et al., revealed an upregulation of genes involved in dead cell uptake (*Timd4*, *Axl*, *Tryo3*), phagosome maturation (*Pikfyve*), lipid and amino acid catabolism, and a downregulation of genes involved in proinflammatory processes (19). Deficiency in, or blockade of dead cell uptake conclusively impacted the polarization of T cells by MPs and DCs and elevated susceptibility to intestinal autoinflammation (154–158). The uptake of apoptotic cells induces transcriptional changes within MPs and DCs that prevent the development of inflammation but also facilitates their functions as gatekeepers of immune cell entry into mucosal tissues. Their role in regulating the entry of immune cells into the gut differs between steady state and inflammation and relies on the ability to interpret different types of cell death and danger signals (159). An elegant study demonstrated that resident MPs are central in recognition of microbial infection and tissue damage to induce the cooperation of resident and newly infiltrating myeloid cells in the bladder. In this study, resident macrophages triggered the chemotactic recruitment of inflammatory monocytes that in turn facilitated the trans-epithelial migration of infiltrating neutrophils across the urogenital epithelium. This process required the release of matrix metalloproteinase (MMP) 9 by infiltrating monocytes (160).

Cell migration into the intestine or across the gut epithelium has been shown to be reliant on matrix metalloproteinases (MMPs) and chemokines. Notably, high expression of MMP-2, MMP-9, MMP-13, and MMP-14 was uniformly reported in all recently identified MP populations (8, 33). MMP-2 in particular has been found to propagate the severity of intestinal inflammation by facilitating the accumulation of infiltrating leukocytes into the colon, while MMP-9 did not participate in this process (161–163). In contrast to the gut, MMP-9 has been demonstrated to facilitate the extravasation of neutrophils across the bladder epithelium, a behaviour also observed during acute intestinal inflammation (160, 164). Whether MMP-9 secretion by gut MPs could facilitate the extravasation of neutrophils across the gut epithelium remains an open question. Interestingly, MMP-8 expression was elevated in blood monocytes and infiltrating leukocytes in the inflamed intestine of IBD patients, suggesting a possible contribution of MMP-8 in the pathogenesis of this disease (144). These studies hint at a possible role for MMPs in the tissue remodelling capabilities of intestinal MPs, especially during inflammation. With data supporting the enrichment of MMPs in MPs within select locations (e.g. MMP-2 and MMP-14 in vasculature-associated MPs), conditional deletion systems should be employed to fully understand the contribution of MP-derived MMPs to gut homeostasis and disease (8).

While the microbiota has not yet been directly implicated in the aforementioned experiments, the close proximity of microbes to the epithelium implies that the gut microbiota may influence epithelial-MP crosstalk. For example, upon bacterial challenge with either *S. pneumoniae* or *E. coli*, CD11c^+^ cells - possibly DCs or a mixture of DCs and MPs -residing beneath M cell-rich areas in Peyer’s patches (PPs) were shown to increase the expression of migration inhibitory factor (MIF), which activated M cell-mediated antigen transport and differentiation (**Figure 2B**) (145, 146). The exact identity of MIF-producing CD11c^+^ cells and a possible involvement of the microbiota in their regulation remain unknown. However, the development of novel human MP-enteroid co-culture models has allowed researchers to study the microbiota-epithelial-MP crosstalk in a more physiologically relevant *ex vivo* setting and will help to shed light into the interactions of gut MPs, DCs and monocytes with epithelial cells (165). Zachos and colleagues utilized this model to demonstrate the ability of villus-residing MPs to extend projections across the epithelium (an observation made in mice before) and phagocytose enterotoxigenic *E. coli*, consequently reducing pathogen load and minimizing epithelial cell death, mirroring the *in vivo* observations in rodents (166). These experimental setups are a fantastic platform for CRISPR/Cas9-based screens to identify MP- and IEC-specific genes involved in the regulation of this important process.

On a body-wide scale, gut-resident eMPs are not the only MP population that adapt to microbial encounters or utilize WNT signaling for wound repair. Following both sterile and infection-induced injury in the lung, *Wnt4*- and *Wnt16*-expressing pulmonary MPs have been demonstrated to induce epithelial proliferation for wound repair. These MPs also produce Trefoil factor 2 (TFF2), a cytokine that initiates mucosal healing. Whether gut eMPs similarly produce TFFs remains an open question, although recent findings show that gut MPs, like those in the lung and skin, produce collagen and platelet-derived growth factor for myofibroblast activation during fibrosis (**Figure 3**) (167–169). However, unlike their lung counterparts, the reparative functions of intestinal MPs are promoted by IL-4 and IL-13, and suppressed by CSF2, suggesting a contextual and microenvironmental regulation of these MP functions (170). Therefore, while new functional questions can be gleaned from comparisons with epithelium-lining MPs in the skin and lung, we should consider that those in the gut may nevertheless have different transcriptional identities, particularly in the context of inflammation.

Indeed, there is still much to be understood about how exactly eMPs contribute to maintaining a homeostatic equilibrium in the intestinal barrier. However, preliminary research alludes to the role of multiple MP-derived secretory factors in this process. Whether it is through removal of dying cells in the gut barrier, modulation of the crypt-villus axis, or regulating the migration of leukocytes, MPs are clearly important in these pathways. More importantly, MPs are also implicated in driving epithelial restitution and wound repair under various inflammatory conditions and at multiple anatomical locations along the gut. Given differential functions across specific anatomical locations, these processes may be driven by the local microbiota or IEC composition.

### Submucosal and LP MPs Support the Enteric Nervous System

The intestinal submucosa and LP house an intricate network of vasculature, lymphatics, and nerves to ensure proper nutrient and immune cell trafficking, host defense against luminal microbes, and peristalsis. Within the LP, immune and mesenchymal stromal cells line the villi and crypts, while lymphoid structures, such as PPs, ILFs, and CPs, serve as hubs for the induction of T and B cells to confer local adaptive immune defense (171).

Recent studies have pointed to the key roles of MPs in maintaining intestinal structural integrity *via* interactions with non-immune cells. These MPs, identified as self-maintaining and long-lived, display distinct transcriptional profiles that correspond to functions specific to their microanatomic location (**Figure 1B**) (8). Similar to the muscularis layer of the intestinal tract, the LP is lined with neurons that are part of the enteric nervous system (ENS) (172). Nerve-associated MPs (nMPs) express genes that have been reported to be enriched in central nervous system (CNS) microglia, such as *Fcrls*, *C1qa*, *Mef2a*, *Gas6*, *Hexb*, and *Gpr34* (8). Interestingly, these nMPs comprise the majority of self-maintaining LP MPs, perhaps reflecting the ability of neurons to confer longevity to myeloid cells. Inducible depletion of long-lived LP MPs in *Cx3cr1* ^CreERT2^ x *Rosa26*-LSL-tdTomato x *Rosa26*-LSL-DTR mice results in increased submucosal neuronal apoptosis and delayed intestinal transit, suggesting that nerve-associated LP MPs are critical in ENS maintenance (8). This observation reveals further similarities between nMPs and CNS microglia, which have been shown to facilitate protection and survival of injured sensory neurons (**Figure 3**) (173, 174).

Nerve-associated MPs undoubtedly exist in other organs as well; for example, CX3CR1^hi^ MPs have been found to associate with sensory neurons in the skin and aid in nerve regeneration after dermal injury or promote photoreceptor homeostasis in the retina (**Figure 3**) (175, 176). Meanwhile, in a model of Wallerian degeneration, long-lived sciatic nMPs were recently described to induce axonal regrowth while newly recruited monocyte-derived MPs engaged in myelin stripping (177). This division of labor between MP populations of different origins may be similarly applicable to the gut - indeed, the role of monocyte-derived gut MPs in maintaining the ENS presents a fascinating outstanding question. Like microglia and Langerhans cells, retina-resident and peripheral nMPs were reported to derive from fetal precursors and partially depend on IL-34 (**Figure 3**) (52, 53, 178). However, it is currently unknown whether gut-resident nMPs require similar survival signals, or whether IL-34 is readily available in intestinal microanatomic niches.

Another fascinating point of similarity between LP nMPs and CNS microglia is their regulation by the microbiota. For example, GF mice display defects in microglia morphology, maturity, and function. In the gut, microbiota-derived signals control both MP turnover rate and the ability to regulate the activity of enteric neurons through muscularis nMP-derived BMP2 (**Figures 1B** and **2C**) (147, 179). These observations suggest a microbiota-MP-neuron axis that may be critical for proper neural functioning, both in the brain and the gut. In concordance with the importance of maintaining a healthy nervous system, enteric neurons themselves have recently been implicated in host defense. For example, Lai et al. described how SI TRPV1^+^ nociceptor neurons directly constrained M cell proliferation through the release of the neuropeptide calcitonin gene-related peptide (CGRP) in order to limit points of entry for *Salmonella*. The limitation in M cell density through nociceptor neurons facilitated the expansion of SFB, a commensal microbe suspected to promote host-resistance to enteric pathogens, which collectively lowered the severity of enteric *Salmonella* infections (180). While the exact mechanism of host-defense remains unresolved, the authors also observed increases in CD11b^+^ CD11c^+^ MHCII^+^ cells (possibly a combination of MPs and DCs) and CD11b^+^ CD11c^-^ MHCII^+^ F4/80^+^ MPs in the PPs of mice treated with the nociceptor agonist, resiniferatoxin, suggesting a possible involvement of nociceptors or nociceptor-regulated M cells in the control of PP myeloid cells (181–183). Meanwhile, Jarret et al. demonstrated that colonic enteric neurons constitutively produced IL-18 to stimulate the secretion of antimicrobial peptides by goblet cells, although it is unknown whether the cytokine affects goblet cells directly or through other mediating IL-18-reponsive cells (181, 182). Whether LP nMPs play a direct or merely supporting role in these pathways is an interesting yet outstanding question that should take the vast heterogeneity of enteric neurons, their location, and their target cells into account (184).

### MPs in the Muscularis Strengthen Neuronal Homeostasis

The intestinal smooth muscle layer, or the muscularis externa, is lined with nMPs that enable intestinal peristalsis (8, 147). Muscularis nMPs display distinctive bipolar and stellate morphology, with two or more pseudopods wrapping around nerve fibers, smooth muscle cells, and fibroblast-like cells (8, 12, 185–187). Multi-photon microscopy from several groups has also revealed a highly patterned mesh-like organization of muscularis-resident nMPs along each layer of the muscularis externa (12, 147). These structural characteristics, along with their relative lack of motility, likely enable optimal nMP-neuronal crosstalk and neuroprotection. Similar to nMPs in the LP, muscularis-resident nMPs regulate the balance of neuronal apoptosis and neurogenesis to maintain a healthy ENS, while enteric neurons in turn maintain nMP survival in a CSF1-dependent fashion (188). Despite their similar ontogeny to nMPs in other organs, gut-resident nMPs express overlapping but also distinct gene signatures, alluding to the possibility of a specific microanatomic gene signature and a tissue-specific gene expression profile (8, 11, 188).

In the SI, nMPs in close proximity to neurons express β_2_ adrenergic receptors (β_2_ARs), which mediate anti-inflammatory polarization *via* neuron-mediated norepinephrine signaling (8). Several genes modulated by the β_2_AR pathway, such as arginase 1 (*Arg1*), have been implicated in neuron survival and axogenesis in other contexts, suggesting a possible feed-back loop between nMPs and neurons (**Figure 2C**) (189, 190). In addition, these nMPs express the neurotransmitter receptor α-7 nicotinic acetylcholine receptor (α7nAChR) and are found in the muscularis externa in close proximity to cholinergic nerve fibers that innervate the vagus nerve. Activation of α7nAChR on these nMPs, likely by neuron-derived acetylcholine, was shown to be required for the vagus nerve-mediated cholinergic anti-inflammatory pathway (CAIP). Experimental electrical stimulation of the vagus nerve, mimicked the CAIP and lowered the expression of *Il1a*, *Il1b*, *Il6* and *Ccl2* in the muscularis externa. Furthermore, activation of α7nAChR through its agonists, nicotine or choline, also blunted the inflammatory response of nMPs to stimulation with extracellular ATP, suggesting a homeostatic neuron-MP axis involved in restraining inflammation (**Figure 2C**) (191). Notably, this study indicates nMPs as a unique intestinal hematopoietic cell type in neuromodulation from brain to gut. While being sustained by enteric neuron-derived CSF1, nMP development was detectable even in the absence of an intact ENS (147, 185). Similarly, aganglionic colons from children with Hirschsprung disease retain normal MP numbers and organization in the muscularis (185). In the absence of enteric neurons, nMPs associate with endothelial cells and interstitial cells of Cajal, two possible compensatory sources for CSF1 (185).

A seminal study from Matheis et al. revealed an intricate interplay between nMPs, enteric neurons, and peripheral neurons involving an Arg1-dependent pathway that protects the ENS after enteric infections (13). Using *Yersinia pseudotuberculosis* and attenuated *Salmonella typhimurium* infections, this group described how muscularis-resident nMPs, but not infiltrating monocytes, limited the NLRP6/Caspase-11-mediated pyroptosis of excitatory VGLUT2^+^ neurons following infection. This long-lasting loss of enteric neurons may account for the impaired GI motility and nerve damage commonly observed in patients suffering from post-infectious irritable bowel syndrome (192–194). Using a series of chemogenetic models and adrenectomy, Matheis et al. demonstrated that systemic stress-driven release of catecholamines by gut-innervating peripheral nerves engaged β_2_AR on nMPs to activate Arg1-mediated polyamine synthesis and secretion, consequently suppressing enteric neuronal NLRP6 and cell death (13). Interestingly, neuronal loss through cell death post-infection was reversible upon transplantation of an intact “pre-infection” microbiota, suggesting a protective role for the microbiota that bears translational potential and requires further investigation (13). Besides the regulation of neuronal cell death, the gut microbiota was shown to facilitate smooth muscle cell contraction through the BMP2-BMPR axis between nMPs and enteric neurons (**Figure 2C**) (147). Together, these studies demonstrate the neuro-protective roles of nMPs both during steady state and enteric infection. Notably, these data further highlight a redundant role for infiltrating monocytes in sustaining enteric neurons and emphasize the importance of the anatomic localization in shaping distinct functions of intestinal MP populations.

The interactions between nerve-associated MPs and tissue cells extend beyond neuro-immune functions and impacts the homeostasis of other intestinal non-immune cells. For example, nMPs are capable of directly interacting with colonic smooth muscle cells using transient receptor potential vanilloid 4 (TRPV4), a sensing channel for thermal, mechanical, and chemical signals. Upon activation, TRPV4 promotes nMPs to secrete PGE2, which activates smooth muscle cells and elicits colonic contraction (**Figure 2C**) (186). Interestingly, cardiac MPs have been reported to electrically modulate cardiomyocytes in a connexin-43- and channelrhodopsin-2-dependent manner suggesting that MPs associated within heavily vascularized tissue may adopt functions of nMP (195). However, experimental proof for this idea has yet to be provided, and it remains unknown whether TRPV4^+^ nMPs utilize gap junction-dependent interactions with muscle cells. This mechanism warrants consideration given reports demonstrating the use of gap junctions between LP MPs and DCs for the transfer of luminal antigens (97). Direct contact between MPs and muscle cells may compensate for peristalsis through neuron-independent pathways to sustain gut motility in the presence of an altered or absent ENS (185). With the microbiome acting as a regulator of nMP functions, studies investigating the role of bacterial biochemical products may open new roads towards a better understanding of how intestinal luminal signals regulate the nMP-neuronal axis. A potential avenue of interest for these investigations could focus on the role of microbiota-produced biogenic amines (**Figure 2C**) (196).

### Intestinal MPs Support the Enteric Vascular System

The second largest group of long-lived LP MPs across both SI and colon are found in close proximity to blood vessels (vasculature-associated MPs, vMPs) and express gene signatures similar to those characteristic of splenic MPs, such as *Adamdec1*, *Apol7c*, and *Dnase1l3* (**Figure 1B**) (8). These MPs form a complex honeycomb lattice structure, with elongations wrapped around the vasculature (35). Adaptation of this structural conformation requires an intact microbiota and serves as a protective shield around the vasculature. In fact, Honda et al. demonstrated that vMPs in antibiotic-treated mice failed to fully wrap the vasculature, resulting in leaky vessels and increased bacterial dissemination (35). The same group further indicated a CCR2-dependent, monocyte-mediated replacement of vMPs in a model of sterile thermal injury in the colon. Intriguingly, the transcriptional factor *Nr4a1*, notably expressed in blood vessel-patrolling Ly6C^lo^ monocytes, was required for the transition of monocyte into vMPs (35, 197). Monocyte-derived vMPs showed a signature gene expression with similarities to Ly6C^lo^ monocytes during the early stages of differentiation, suggesting a role for Ly6C^lo^ monocytes as an origin of intestinal vMPs. Further comparisons between intestinal vMPs and vasculature-patrolling Ly6C^lo^ monocytes may yield interesting insights into the ontogeny of this understudied MP population. Interestingly, in the thymus, *Nr4a1* has been reported to control the development of a CD11b^-^ F4/80^+^ Tim-4^+^ MP population that displays superior phagocytic capacity and contributes to the clearance of apoptotic cells (**Figure 2D**) (198). While these findings imply that *Nr4a1*-dependent Ly6C^lo^ monocytes may differentiate into vMPs, direct experimental evidence is lacking. Given that the adoptive transfer of Ly6C^hi^ monocytes generates gut MPs and that Ly6C^hi^ monocytes infiltrating the heart and lung express low levels of *Nr4a1*, an Nr4a1-dependent transcriptional adaptation of MPs, once located in proximity of vascular endothelial cells, may drive the differentiation of monocytes into vMPs (37, 180, 199–202). However, whether this hypothesis is true for vMPs in the gut remains to be formally validated.

Nerve- and vasculature-associated LP MPs have both been implicated in the maintenance of their respective neighboring cells, as inducible depletion of these long-lived LP MPs (including nMPs and vMPs) in *Cx3cr1* ^CreERT2^ x *Rosa26*-LSL-tdTomato mice results in increased submucosal neuronal apoptosis and vascular leakage (8). Although we have yet to identify unique surface markers to distinguish these MP subsets from other populations, the lymphatic marker and hyaluronan receptor LYVE-1 may serve as a possible candidate for vMPs. In humans and mice, arteries are lined with LYVE-1^+^ MPs that modulate collagen expression by smooth muscle cells *via* LYVE-1-hyaluronan interactions to maintain appropriate arterial homeostasis (203). A subset of cardiac MPs co-expressing LYVE-1 and Tim-4 has been reported, while interstitial MPs in the lung, fat, heart, and dermis were found to contain two distinct populations, one being LYVE-1^hi^ MHCII^lo^ and associated with blood vessels and a LYVE-1^lo^ MHCII^hi^ nerve-associated MP population (34, 204). Considering this consistency across organs, gut-resident vMPs and nMPs may adopt a similar surface marker expression profile given their localization within the tissue. Due to the tight associations between the intestinal vasculature and nerves, it is possible that there are shared functions between vMPs and nMPs. For example, MPs associated with cardiomyocytes can facilitate electrical conduction at a highly vascularized site (195).

When referring to the vascular system of the intestine, it is noteworthy to mention the involvement of LP MPs in regulating the maturation of the lacteal, a long, blunt-ended, tube-like lymphatic vessel located in the center of each intestinal villus (**Figure 2D**). These structures play an important role in the absorption of dietary lipids in the small intestine and require CX3CR1^+^ MPs for appropriate elongation and integrity, suggesting a supportive role for gut MP in lymphatic vessel homeostasis. Interestingly, microbiota-derived cues initiated the production of vascular endothelial growth factor (VEGF)-C in gut MPs, a growth factor supporting the integrity and length of lacteals (**Figure 2D**) (205). In turn, a role for lymphatic endothelial cells in supporting subcapsular sinus MPs has been reported. Mondor et al. demonstrated that the production of CSF1 by lymphatic endothelial cells constitutes a crucial niche for maintaining subcapsular sinus MPs during embryonic and adult life (206). Whether an interaction like this, either directly or through an indirect crosstalk *via* the lacteal-underlying stromal compartment, feeds back into the maintenance of vMPs in the gut remains an open question (**Figure 3**).

Stromal cells have long been neglected and seen as a passive structural entity in tissues. However, a growing body of research has begun to support historic findings that indicated an active role for stromal cells in shaping the intestinal immune system (207). For example, LPS stimulation of intestinal fibroblasts and myofibroblasts triggers the production of CSF1 and CSF2, both required for myeloid cell survival and differentiation (208, 209). More recently, Kim et al. demonstrated that LP stromal cells, but not epithelial cells, secreted CCL2 in a NOD2-dependent manner during *C. rodentium* infection to recruit monocytes (210). A different group further suggested that BM-derived mesenchymal stromal cells can secrete CCL2 and CXCL12, subsequently upregulating IL-10 expression in CCR2^+^ MPs in a model of chemically induced colitis (211). In other organs, such as the liver, peritoneal cavity, and even joints, local stromal cells have been shown to play critical roles in shaping tissue-resident MPs as well (212, 213). Unfortunately, not much is currently known about how MPs may in turn affect stromal cells in the intestinal tract. Given their role in guiding immune cell localization, intestinal stromal cells may be vital in orchestrating the microanatomic location of LP MPs. However, at this point we are merely speculating and urging the need to further explore this intricate crosstalk. The yielded observations will provide exciting insights into MP-stroma cell interactions, particularly in the context of a complex organ such as the intestinal tract.

## Translating Murine MP Biology Into Humans

Multiple limitations hinder the ability to effectively study MP heterogeneity in the human GI tract. These challenges include the enormous size of the organ, limited tissue accessibility, and confounding factors that impact immune system development, such as demographics, lifestyles, diets, medications, and the microbiota. Frequent biopsy sampling during the monitoring of the intestinal tract and the often disease-associated requirement for tissue resections in IBD patients offer an opportunity for scientists to overcome the hurdle of tissue availability. However, large scale genome-wide association studies and high-dimensional single cell analyses have substantially enhanced our understanding of human gut MP heterogeneity particularly during IBD. Two comprehensive summaries of the current understanding of MPs in IBD can found elsewhere (214, 215).

As in mice, human MPs arise through distinct waves of myelopoiesis seeding early developing organs (216). Bujko et al. published a landmark paper describing the identification of at least 4 distinct MP populations (termed Mf1-4) in the small intestine of humans. In line with previous studies in mice, MP subsets expressing high levels of CD14 and CCR2 (Mf1 and Mf2) displayed rapid replacement by infiltrating recipient monocytes in small bowel transplants (217). Recruitment of monocytes to the human colonic mucosa has been shown to require CCR2 (218). Collectively, these findings suggest that monocytes are precursors of MPs in the human SI and colon (217). Similar to the observations made by Liu et al. and De Schepper et al., long-lived MPs in the human SI (Mf3 and Mf4) showed a delayed turnover rate but were fully replaced within one year post organ transplantation (8, 36).

In line with reports on murine models of IBD, a pro-inflammatory monocyte-like intestinal MP subset, defined as CD45^+^HLA-DR^+^CD14^+^CD64^+^CD11c^high^CCR2^+^CX3CR1^+^, was found to accumulate in the inflamed colonic mucosa of IBD patients (37, 38, 218). High-dimensional single cell analysis of the HLA-DR^+^CD172a^+^ cells (containing MPs and some DC subsets) in the inflamed colonic mucosa identified two CD14^+^CD64^+^ MP subsets that are discriminated by their CD163 expression. Interestingly, the CD14^+^CD64^+^CD163^-^ MP population was selectively increased in the inflamed tissue of CD patients and suspected to contribute to inflammation by fostering the polarization of Th17 cells, as demonstrated *in vitro* (219–221). In contrast, a CD14^+^ CD64^+^ CD163^+^ CD160^-^ MP subset was reported to promote Th17 cells in UC patients, emphasizing the complex interplay between myeloid cell function and IBD (219). Similarly, a CD14^+^ population expressing both MP (CD33, CD68) and DC (CD205, CD209) markers has been described in the LP and was found to be increased in CD patients (222, 223). Stimulation of these cells with two distinct commensal bacteria, *Escherichia coli* and *Enterococcus faecalis*, led to significantly increased secretion of pro-inflammatory mediators, including IL-12/IL-23p40, IL-23, TNF-α, and IL-6 (222, 223). In a more recent study, colonization of germ-free mice with IBD microbiotas was shown to alter the T cell landscape and adoptive transfer of these T cells into DSS-treated *Rag1* ^-/-^ mice conferred increased disease severity (222, 223). Taken together, these findings implicate a role for the human gut microbiota and immune cell crosstalk in intestinal inflammation (222, 223). Local cytokine signals affect human gut MP functions as well. Mutations in *CSF2RB* and *IL10R* recapitulate the reports in mice of altered MP homeostasis and confer increased disease susceptibility to IBD (115, 224–226). Single-cell mRNA analysis of the inflamed and non-inflamed CD mucosa identified a cellular module comprised of plasma cells, inflammatory MPs and DCs, activated T cells, and stromal cells within ileal lesions (128). An increase in inflammatory monocytes and MPs was shown to be a hallmark of this CD-associated module and is suspected to initiate an inflammatory cascade involving stromal cell-mediated recruitment of inflammatory adaptive immune cells (128). Importantly, the presence of this module correlated with non-response to anti-TNF treatment. This study demonstrates the need to further characterize human gut MP subsets and possible interactions with intestinal cells in order to improve treatment strategies for IBD patients. Collectively, these reports have uncovered parallels between human and murine MP development and function, thus validating the use of mouse models to inspire translational studies.

## Concluding Remarks and Future Outlook

In comparison to our current knowledge of tissue-resident MPs in other organs such as liver, lung, or brain, there is still much unknown about intestinal MP heterogeneity (**Figure 3**). Significant progress in identifying unique populations of gut-resident MPs and the insights gleaned from these studies are starting to reveal the complex and nuanced structure of each unique MP niche (**Figures 2** and **3**). However, key outstanding questions remain. For example: What is the cellular composition of MP niches in the intestinal tract? Which factors regulate each MP population within their intestinal microanatomic location? How plastic are gut MPs within their respective micro-environment? Are MPs travelling between microanatomic locations of the gut and would this affect their respective phenotype? How do changes in tissue cell composition or activation affect MP heterogeneity within their local niches? Molecular and cellular interactions of tissue cells and MPs in the liver, spleen, lung, heart, or brain should inspire the analysis of homologous niches and roles for gut-resident nMPs, vMPs, eMPs, and lMPs (**Figure 3**). Further research into the heterogeneity of intestinal MP should focus on the characterization of their distinct transcriptional profiles and integrate the regulation of these networks into the local microenvironmental context. Dissecting this heterogeneity is necessary to improve our understanding of the importance of gut-resident MPs in tissue maintenance, development, repair, host defense, and microbial tolerance. This undertaking will capture the full diversity of tissue-MP interactions across the entire intestinal tract and could shed light on why some gastrointestinal diseases (e.g. CD or UC) affect defined regions of the gut.

## Author Contributions

PC, SLT, LN and AM wrote the manuscript and designed the figures. All authors contributed to the article and approved the submitted version.

## Funding

PC is supported by an Ontario Trillium Scholarship and a Vanier/NSERC Canada Graduate Scholarship. LN is supported by an Ontario Graduate Scholarship. SLT is a recipient of the Dr. Edward Ketchum Graduate Student Scholarship and the Canada Graduate Scholarships – Master’s (CGS M) award. AM is supported by a CIHR-Project Grant (388337) and a NSERC-Discovery Grant (RGPIN-2019-04521). AM is the Tier 2 Canadian Research Chair in Mucosal Immunology and supported by the Tier 2 CRC-CIHR program.

## Conflict of Interest

The authors declare that the research was conducted in the absence of any commercial or financial relationships that could be construed as a potential conflict of interest.

## Publisher’s Note

All claims expressed in this article are solely those of the authors and do not necessarily represent those of their affiliated organizations, or those of the publisher, the editors and the reviewers. Any product that may be evaluated in this article, or claim that may be made by its manufacturer, is not guaranteed or endorsed by the publisher.
